# The role of link workers in weight management for people with severe mental illness: a qualitative study

**DOI:** 10.1186/s12875-025-02929-4

**Published:** 2025-08-09

**Authors:** Rija Imran, Alexandra Kenny, Geoffrey Wong, Charlotte Lee

**Affiliations:** 1 School of Clinical Medicine, University of Cambridge, Cambridge, Cambridgeshire, CB2 0SP UK; 2 The McPin Foundation, Unit 1.4, The Green House, 244-254 Cambridge Heath Road, London, E2 9DA UK; 3 Nuffield Department of Primary Care Health Sciences, University of Oxford, Oxford, Oxfordshire OX2 6GG UK

**Keywords:** Primary health care, Link workers, Weight reduction programmes, Schizophrenia spectrum and other psychotic disorders, Qualitative research

## Abstract

**Background:**

People with severe mental illness (SMI) have an increased risk of cardiovascular disease, partly due to factors such as overweight and obesity. Weight management programmes can potentially reduce this risk, but people with SMI face barriers to access and engagement.

**Aim:**

To explore the acceptability of using link workers to address barriers to accessing and engaging with weight management programmes for people with SMI.

**Design and Setting:**

A qualitative study conducted with people with SMI, link workers, and health promotion workers in primary care. The study was co-designed and co-delivered with a person with lived experience of SMI.

**Method:**

Five online focus groups and dyad interviews were run with 13 participants, including seven people with SMI and six health professionals (three link workers and three health promotion workers), between 25 July 2023 and 31 March 2024. Discussions were audio-recorded, transcribed verbatim, and analysed using a codebook thematic analysis in NVivo software.

**Results:**

We constructed three analytical themes. (1) The view of link workers: all participants saw link workers as valuable in overcoming emotional and practical barriers to weight management. (2) Expectations of link worker support: support must be personalised, culturally responsive, and focused on building trust. (3) Challenges for link workers: barriers included limited mental health training, undefined roles, and capacity concerns.

**Conclusion:**

Link workers offer an acceptable, low-intense approach to improving access to weight management programmes for people with SMI. Strengthening coordination between link workers and weight management services in primary care, as well as defining their role and level of support, could improve outcomes for this underserved group.

**Supplementary Information:**

The online version contains supplementary material available at 10.1186/s12875-025-02929-4.

## Introduction

People with severe mental illness (SMI) have a life expectancy 15 to 20 years shorter than the general population [1, 2], with cardiovascular disease (CVD) accounting for two-thirds of this premature mortality [3]. Excess adipose tissue is a key modifiable risk factor for hypertension, dyslipidaemia, type 2 diabetes, and therein CVD [4]. This underscores the pressing need to address adiposity in people with SMI. While antipsychotics are commonly used to manage the symptoms of SMI, they are known contributors to weight gain [5]. The notion, however, that excess weight is inevitable ought to be challenged.

In the United Kingdom (UK), tier 2 weight management programmes are the mainstay treatment for excess weight, often offered free at the point of need in primary care [6, 7]. Randomised controlled trials (RCTs) have consistently shown that people with SMI can achieve meaningful weight loss by participating in these weight management programmes that intervene on diet and physical activity. One systematic review of 34 RCTs testing 36 interventions in 4,305 participants observed that active interventions resulted in more weight loss (mean = −4.37 to + 1 kg at 6 weeks to 18 months follow-up) compared with control groups (−1.64 to + 3.08 kg) [8], which is commensurate with observations in the general population at one-year follow-up [9]. Annual health checks for people with SMI, such as those delivered in primary care by NHS England, offer a potential entry point for referring people with SMI and excess weight to such programmes [10]. 

Despite the projected benefits of weight management programmes, people with SMI have reported specific barriers to accessing and engaging with them. These include fears about initiating social interactions rooted in concerns about panicking, negative judgements, or what other people or voices might do or say [11]. Difficulty concentrating, fluctuations in mood, low self-compassion, and high self-criticism can also can hinder weight loss attempts [12]. Furthermore, people with SMI face systemic barriers to accessing primary health care, including public, institutional, and anticipated stigma, as well as fragmented services, which compound challenges in attending weight management programmes [13]. Bespoke programmes can address some of these barriers, but they are rarely offered in primary care because a cost-effectiveness analysis has indicated they are not a viable option [14]. A more sustainable approach may involve wrap-around support to address these emotional and practical challenges, enabling people with SMI to benefit from existing weight management programmes [15]. 

Link workers– also known as social prescribers, community connectors, or community navigators– are non-clinical practitioners based in primary care. The role is designed to support people with social and practical issues that affect their health and wellbeing, while improving general practitioner time and efficiency [16]. Operating within the social prescribing pathway, link workers connect people to community resources, such as walking groups, luncheon clubs, and art-based activities. The NHS Long Term Plan commits to expanding the link worker role from over 3,000 positions in 2022 to 9,000 by 2036/37, to provide support alongside a cadre of primary care staff, including health promotion workers, community mental health nurses, and clinical pharmacists. In this context, link workers may be well positioned to help people with SMI access and sustain participation in weight management programmes as a preventative measure for CVD. However, there is little evidence on how accepted they are in this role or whether other staff might provide alternative or complementary support. 

This study aimed to explore the acceptability of using link workers to support people with SMI access and engage with tier 2 weight management programmes. Our objective was to consult both people with SMI and link workers to assess the barriers and facilitators of this approach and its potential to improve access and engagement.

## Methods

### Design and reporting

This was a qualitative study involving focus groups and dyad interviews with people with SMI and link workers. We followed the Consolidated Criteria for Reporting Qualitative Studies (COREQ) criteria to report it (see Supplementary Data S1) [17, 18]. 

### Patient and public involvement and engagement

The study was co-designed in collaboration with a patient and public involvement and engagement (PPIE) champion who had a lived experience of SMI. A research psychologist, with an academic interest in weight management for people with SMI, co-designed a semi-structured topic guide with the PPIE champion, which was piloted with an external member of the research team. The topic guide included 10 open-ended questions, with probes to scaffold participants’ responses or to explore comments relevant to the research question (see Supplementary Data S2). Focus groups and dyad interviews were co-facilitated by the researcher and PPIE champion, who received training to lead these discussions.

### Sampling procedure and recruitment

People with SMI (i.e., community, inpatient, or both) were recruited through convenience sampling via the McPin Foundation– a charity that puts the lived experience of people with mental illness at the heart of research (www.mcpin.org). An online advertisement was posted on the McPin website and anyone interested could directly contact the study team email. Interested participants received an information sheet, followed by a telephone screening call to assess their eligibility, obtain informed consent, and collect demographic data. Two participants were already known to the research team through previous involvement in unrelated studies, but had no ongoing therapeutic or professional relationships with us at the time of recruitment.

The eligibility criteria included: aged 18 or older; given a primary diagnosis of SMI (i.e., ICD-10 codes F20-39 including schizophrenia, schizophreniform disorder, schizoaffective disorder, delusional disorder, brief reactive psychosis, or psychosis not otherwise specified, and bipolar disorder); living with overweight (body mass index [BMI] 25–29.9 kg/m2) or obesity (BMI ≥ 30 kg/m^2^); willing and able to provide informed consent; and access to Microsoft Teams.

Link workers were also recruited through convenience sampling via the Social Prescribing Research Network– a network that includes members of the public, clinical providers, the voluntary community sector, and policy makers (www.socialprescribing.phc.ox.ac.uk). An online advertisement was emailed to people who had subscribed to the network’s newsletter. As with the SMI group, interested participants were given an information sheet, and invited to a telephone call to assess their eligibility, obtain informed consent, and collect their demographic data.

The eligibility criteria included: aged 18 or older; working in a community-based role in England; willing and able to provide informed consent; and access to Microsoft Teams. There was no exclusion on seniority or length of experience in their role.

While participants were not explicitly asked to recruit through word of mouth or their own networks, any referrals made by them were welcomed by the research team. Recruitment continued until we reached theoretical data sufficiency or the end of the 8-month study period, whichever came first.

### Data collection

All focus groups and dyad interviews were conducted remotely as a co-design feature, advocated by the PPIE champion, to promote accessibility and reduce participation burden, especially for people with SMI who have reported mobility issues and concerns about judgement or harm from others. Our decision to run separate focus groups was also informed by the PPIE advice during our study design phase, who indicated that people with SMI might prefer to engage in small group discussions with others who share their lived experience. Additionally, conducting small groups discussions of this size allowed greater flexibility to explore consensus (or lack of) agreement among the participants. 

To our knowledge, only the researcher(s) and interviewee(s) were present during the discussions. All discussions were recorded on a separate audio device and transcribed verbatim. No field notes were taken, but the researcher and PPIE champion debriefed after each focus group they co-facilitated. Focus groups and dyad interviews were scheduled for up to one hour with one five-minute break and took place between 25 July 2023 and 31 March 2024. All participants were renumerated with a £25 per hour voucher for their participation. There were no repeated focus groups and transcripts were not returned to the participants.

### Data analysis

We followed an inductive-deductive codebook thematic analysis since we aimed to learn about and describe phenomena which little is known ahead of designing an intervention [19, 20]. We did not intend to generate new theories or concepts, but rather explore how all participants accepted the link worker role.

This analysis followed the following steps. First, we deductively developed a priori codes based on our PPIE input and previous research to create an initial coding framework [15]. Second, two researchers read and re-read the transcripts to identify and discuss potential codes, revising the initial coding framework where necessary. Third, the revised coding framework was applied to the interview transcripts. In this step, we attached labels to meaningful units of text in each transcript and inductively-deductively coded them under the coding framework. Fourth, the study team met to discuss the data coding and interpretation, make further iterations to the framework, and reach a consensus agreement. Fifth, once all transcripts had been (re)read and (re)coded, they were electronically managed, which allowed us to easily visualise the data and produce a codebook. The codebook was read and re-read to identify possible patterns between and within interviews, and to identify deviant cases. Sixth, codes were organised and re-organised into sub-themes and then descriptive themes to produce top-level analytical themes.

We acted on Miles and Huberman’s recommendations to enhance trustworthiness in our findings [21, 22]. This meant we used data triangulation, which involved analysing data from multiple sources, namely people with SMI, link workers, and health promotion workers, to account for any convergence and divergence in their perspectives. We used investigator triangulation by engaging in regular discussions and scrutinising the researcher’s reflexivity log to maintain rigour. We also applied theoretical triangulation and sought the PPIE champion’s feedback on the findings to ensure our interpretations remained close to their own account. Our ontological position was relativism, and our epistemological stance was rooted in subjectivism. All data were managed in NVivo 1.7.2 software [23]. 

## Results

### Demographic characteristics

In total, 13 participants took part: seven people with SMI, three link workers, and three health promotion workers. Our decision to include health promotion workers was based on whether they could offer alternative or complimentary support to link workers in assisting people with SMI. We ran five sessions in total: one focus group with three people with SMI, two dyad interviews with two people with SMI each, one dyad interview with two link workers, and one focus group with four health promotion workers. The sessions lasted on average 64.8 minutes (range = 61 to 71 minutes). No participants refused to take part or withdrew from the study.

Participants with SMI had a mean age of 43.5 years. Of the seven participants with SMI, three were female, three were white, three were Asian, and one was of mixed ethnicity. Four had schizophrenia spectrum disorder while three had bipolar disorder. We did not ask participants with SMI if they were actively trying to lose weight, but all reported living with overweight or obesity, and none had prior experience working with a link worker. Among the link and health promotion workers, the mean age was 42.5 years. All six were female, five were white and one was Asian, and they averaged 2.3 years of professional experience.

We triangulated the views of people with SMI, link workers, and health promotion workers and constructed three analytical themes: (1) the view of link worker support; (2) expectations of link worker support; and (3) challenges for link workers. Selected quotations are given below, and personal and brand names have been pseudonymised. Additional quotes are provided in the Supplementary Data S3.

## Themes

### Theme 1: the view of link workers

#### Overcome initial fear and anxiety

Many people with SMI reflected on how their world had changed since their diagnosis, making once familiar activities seem entirely new. This acted as a barrier to joining a weight management programme, which required openness and vulnerability to step into a fearful unknown. One person with SMI likened the experience to a child trying something on their own for the first time and questioned why more support was not available for people in such vulnerable positions.

*“it’s the actual experience that I went through that made living [um] very scary. So [um] you’re coming out of a very scary place and then you’re trying to find your place in the world again*,* and everything feels new. I can’t*,* I can’t*,* I can only*,* yeah it’s like you’re doing it for the first time again.”*– Janice, 54-years old female, diagnosed with schizophrenia.

Most participants across all groups said that taking the first step into a new setting required more than personal resolve– it needed external encouragement. In this context, the link worker role was seen as serving an important function; it held space to reassure people with SMI of their valid concerns and to build confidence initiating a weight loss attempt.

*“… having somebody who’s like a link worker just to build you that confidence up to go and do it*,* and then just continue*,* continually basically maintain losing the weight*,* if that makes sense.”*– Ivan, age not given, male, diagnosed with bipolar disorder.

#### Foster a sense of belonging

Some people with SMI shared their sense of being ‘othered’, fuelling a feeling of not belonging, which made some of them feel out of place at weight management programmes. These participants expressed a need for advice to help manage isolating feelings and make weight loss groups seem more approachable.

Others viewed the link worker role as a valued chance to build a therapeutic alliance. This went beyond advice; it was about feeling genuinely understood in their lived experience. For some people with SMI, having someone recognise the impact of their diagnosis and the challenge of re-entering the world renewed their self-belief.

*“I think it’s that therapeutic relationship*,* like I said with the GP. Somebody who*,* who*,* who believes in you and*,* and helps you develop that courage.”–* Ivan, age not given, male, diagnosed with bipolar disorder.

#### Offer practical support

Some people with SMI said poor transportation prevented them from accessing weight management programmes. While surmountable, this was viewed as a significant obstacle for people with SMI. In another instance, one participant described how medical restrictions prevented them from driving.

*“And travelling on a bus is very*,* is*,* is okay but it’s a bit anxious.”*– Amir, 42-years old male, diagnosed with psychosis.

Link workers were viewed as a person to facilitate transportation, and one person with SMI said they wanted link workers to accompany them to the weight management programme. While some link workers acknowledged that this is sometimes within their remit, others pointed out that this type of support varies across primary care networks and insurance coverage. In addition, there was uncertainty about who should initiate this support, as both groups relied on each other to raise this type of offer.

*“… take us there and you know maybe get us introduced to stuff like that.”*– Adnan, 26-years old, male, with schizoaffective disorder.

*“you know if they bring up that they want to lose weight then you know I will talk to them about the options*,* signpost*,* [um] offer support*,* access groups*,* [um] make referrals for them*,* [um] be someone who’s encouraging them [um] and trying to sort of motivate them.”*– Clara, 57-years old, female, link worker.

### Theme 2: expectations of link workers

#### Provide an accountable space

Most participants across all groups valued assertive but supportive communication to stay on track of weight loss goals. Some participants highlighted that a check-in (e.g., brief telephone calls or emails) were a way to enact this support and could even help reduce feelings of isolation for people with SMI.

*“…if I can do something that makes them not feel as isolated it might also help them to motivate to then to go out and do things.”*– Jade, 39-years old, female, link worker.

Most participants emphasised support be consistent throughout the programme, not just at the beginning. One person with SMI said that without ongoing support, they would sometimes “mak[e] excuses” to avoid anxiety-inducing situations altogether.

*“It doesn’t have to be forever*,* but just*,* it’s just*,* I think recognising where*,* where a person has been through*,* what*,* what a person’s been through when they’ve been through psychosis.”*– Janice, 54-years old female, diagnosed with schizophrenia.

#### Be personalised and culturally competent

All participants recognised the need for flexibility when working with people with SMI, as their circumstances often change due to their illness. Consistency and dependability through both ups and downs would help build the therapeutic alliance.

*“I think working with people with severe mental illnesses also requires a level of flexibility and how you are willing to support them in a way*,* because sometimes their situation might change and if you then go*,* “Okay*,* when*,* well then since it’s changed it’s now doesn’t fit in with what we do. You can come back to us when your situation changes back to how we can support you.”*– Elizabeth, 46 years old, female, health promotion worker.

People with SMI emphasised the need for cultural competency, as conventional suggestions like joining walking groups or coffee mornings could be irrelevant for people from different backgrounds, languages, or physical abilities.

*“… well English is probably about her fifth language*,* so there’s no way she’s going to join a coffee morning group with what will end up being a group of white women*,* white British women… So it was just*,* it was totally pointless.”–* Adnan, 26-years old, male, diagnosed with schizoaffective disorder.

### Theme 3: challenges for link workers

#### Offer specialised knowledge and training

All participants highlighted the importance of appropriate mental health training for those providing the wrap-around support. It was deemed important to equip any support provider with knowledge on the unique challenges faced by people with SMI and skills to garner trust.

*“In terms of severe mental illness*,* in terms of like sort of supporting people with bipolar or schizophrenia or whatever it is*,* that is probably something that is a bit of a gap and we don’t necessarily have that training”*– Corinne, 35 years old, female, heath promotion worker.

#### Define the role of link workers

Link workers and health promotion workers shared concerns that whilst they want to be flexible, lack of clear boundaries in their role meant some patients could become dependent on them. Some expressed ambiguity about the specific role of link workers or how they could define their boundaries.

*“I’m not going to say to someone*,* “Well we’re done now*,* your six months are up*,*” if it’s still making a difference… [but] my job is to empower them not to become a crutch for them.”–* Jade, 39-years old, female, link worker.

*“you just wonder about whether people have first of all the time*,* because if a social prescriber is then going to be almost like a doctor*,* it would be ten minutes per problem*,* and it’ll be kind of like well you know*,* “Well we can’t fit all of your life history and your emotional drama right now. Why don’t you book another appointment?”*– Rukshana, 45 years old, female, bipolar disorder.

#### Address capacity limitations

All participants recognised that link workers are valued but limited in their availability due to high demand. People with SMI said they want consistent support, especially in the early stages of joining a weight management programme, to build rapport and maintain motivation. However, link and health promotion workers emphasised that large caseloads meant they felt unable to provide the level of support desired.

*“you know like I can have a hundred people on my caseload*,* I haven’t got capacity to talk to someone every week*,* [um] and come up with those different approaches and think of things.”–* Clara, 57-years old, female, link worker.

*“I’m the only social prescriber in my *PCN [primary care network],* you know that’s seven practices across quite a large geographical area*,* and I go out and do home visits for most people initially*,* [um] but we have two health and wellbeing coaches for the same locality*,* so it’s a capacity issue [um] of who’s able to do that?”–* Jade, 39-years old, female, link worker.

## Discussion

This qualitative study explored how link workers could improve access to and engagement with existing tier 2 weight management programmes from the perspectives of people with SMI, link workers, and health promotion workers. Participants with SMI highlighted a need for emotional and practical support and suggested that link- and health promotion workers could help them overcome barriers such as fear, anxiety, and social isolation. However, participants across all groups noted insufficient mental health training, undefined boundaries, and capacity concerns. Nonetheless, adjunctive support from a link worker alongside a primary care referral to a weight management programme was valued for its potential to deliver personalised and culturally-sensitive care, and foster confidence to sustain weight loss efforts among people with SMI.

### Strengths and limitations

We provide insights from key stakeholders to refine an approach for maximum relevance and acceptability ahead of feasibility testing [24]. We integrated PPIE throughout the research process, which meant we co-designed the study, co-designed the topic guide, co-facilitated the focus groups and dyad interviews, and co-reviewed findings with a PPIE champion. This helped to ensure the research was informed by the lived experience of SMI and was relevant to their priorities. Ethnic minority groups were well-represented, comprising 57% of our group with SMI, which allowed us to capture diverse perspectives and helped ensure that our research was culturally relevant. The topic guide was semi-structured, which provided flexibility, allowing us to probe the participants to discuss pertinent opinions and experiences. The focus group and dyad interview method likely created more balanced power dynamics and fostered a safe, trusting environment that encouraged openness and authentic engagement.

The recruitment strategy had limited reach because it relied on conveniently sampling via traditional methods, such as online advertising and newsletter mailouts. This approach may have introduced self-selection bias, attracting people who are more motivated and engaged in research while potentially excluding those who may lack access or awareness of such opportunities [25]. Furthermore, all participants were eHealth literate and had access to digital technology, which could affect the transferability of the findings. We were unable to recruit our projected number of link workers within the study timeframe, despite repeated advertisements. This may reflect competing demands on link workers’ time, workforce pressures in primary care, or a lack of perceived relevance of the study to their day-to-day responsibilities [26]. Furthermore, we did not pursue additional recruitment strategies. This is not inherently problematic in qualitative research because the goal is to reach theoretical sufficiency — the point at which data adequately supports the research objectives. In our study, we achieved theoretical data sufficiency, supporting the transferability and clinical utility of our findings [25]. The focus group and dyad interview setting could have introduced respondent bias, where participants altered their responses to conform to others, although we tried to minimise this by keeping the groups small (*≤* 4 participants) [25]. Another point is that no one with SMI reported having experience with a link worker, which may have influenced their perspectives due to their unfamiliarity with this type of support. While both interviewers were trained in qualitative methods and all researchers contributed to the data analysis, researcher bias cannot be entirely excluded. However, our use of reflexive practices encouraged us to acknowledge our own assumptions, experiences and opinions. This was a recursive process to enhance trustworthiness [19]. 

### Comparison with existing literature

Weight management programmes remain the mainstay intervention to address excess weight in people with and without SMI. Alternative strategies — such as antipsychotic medication optimisation, cognitive-behavioural therapy, and even peer support — offer complementary benefits and function within a suite of options [27]. However, these alternatives are not widely implemented in primary care, and research comparing their effectiveness against standard weight management programmes is limited.

Despite the availability of weight management programmes, people with SMI often encounter barriers to accessing them. Social support– defined as emotional, informational, and practical help from one’s network– is a predictor of weight loss in people with SMI [28]. It can be delivered by family members, friends, and peer supporters [29], but many with SMI have reported unreliable support due to strained relationships or social isolation [30]. This study focused on link workers as one viable source of support within the primary care cadre of staff, as their role is designed to connect people in primary care with existing community resources.

Most participants highlighted the value of link workers to support people with SMI to initiate and sustain weight management efforts. Essential to this support was the link worker’s knowledge of mental illness, strong communication skills, and the willingness and capacity to serve as an anchor point. This finding is consistent with a realist evaluation on the concept of ‘holding’ among link workers, defined as maintaining a trusting, supportive, and therapeutic relationship without the expectation of a cure [31]. It allows space for the patient and link worker to consider social problems that affect health, and better support patient-led decisions [31]. While holding space is accepted as a function of the link worker role, prioritising their training to do this will be necessary for staff well-being and positive outcomes for the patient [26]. 

Participants noted that link worker support could extend beyond weight management to address broader issues such as social, economic, or cultural barriers. Regular and sustained support was seen as essential for overcoming setbacks and making progress with health-related goals. This finding is congruent with previous qualitative studies highlighting the wider socio-economic challenges reported by people with SMI in weight management, including feelings of isolation and stigmatisation [32] and a lack of long-term support [33]. However, this study and meta-ethnographies suggest that link workers often face stress stemming from ambiguity in their role and undefined boundaries [34]. To leverage link workers in supporting access to weight management programmes for people with SMI, it will be necessary to provide clear role definitions.

### Implications for research and practice

There is pressing action needed to address the premature mortality in people with SMI. The NHS Long Term Plan has renewed its commitment to tackling this health inequality by targeting CVD prevention [35]. Weight management programmes show promise in supporting this agenda, yet barriers to access and engagement remain unaddressed for people with SMI. Leveraging link workers to provide low-intensity, wrap-around support offers a potentially novel and acceptable approach to address the unique needs of people with SMI and might represent good value for the public purse.

NHS England’s universal personalised care model outlines plans to expand the link worker role over the next decade [36]. This offers an opportunity to provide additional support alongside a referral to a weight management programme in primary care. However, our data indicate that people with SMI may seek support that extends beyond weight loss, including assistance with socio-economic and emotional factors, such as poor housing, economic hardship, and isolation [34]. The broader context of health and social care systems in many high-income countries further compounds these expectations. While link workers are generally equipped to address such issues, they are finding themselves increasingly limited in their availability due to high demand and unclear role definitions [34]. As a result, link workers may have scepticism regarding their capacity to take on additional responsibilities, such as supporting weight management for people with SMI.

## Conclusions

Link workers offer an acceptable, low-intensity approach to improving access to weight management programmes for people with SMI. Strengthening coordination between link workers and weight management services in primary care could improve outcomes for this underserved group. Defining the level of support and ensuring specific mental health training for link workers will be essential for its success.

## Supplementary Information

Supplementary Material 1.

## Data Availability

The data generated or analysed during this study are available from the corresponding author on reasonable request.

## Abbreviations

COREQ: Consolidated Criteria for Reporting Qualitative Studies
CVD: Cardiovascular disease
NHS: National Healthcare Service
PPIE: Patient and Public Involvement and Engagement
RCT: Randomised Controlled Trial
SMI: Severe Mental Illness
