# Supplementary material for: The role of link workers in weight management for people with severe mental illness: a qualitative study
Source: BMC Prim Care. 2025 Aug 9;26:251. doi: 10.1186/s12875-025-02929-4 (PMC12335018; doi:10.1186/s12875-025-02929-4)
Supplement: Supplementary file 1 — Supplementary Material 1. [file 12875_2025_2929_MOESM1_ESM.docx]

**The role of link workers in weight management for people with severe mental illness: a qualitative study**

# **Supplementary Data**

**S1. The COREQ checklist**

| **Item number** | **Item** | **Description** | **Reported** |
| --- | --- | --- | --- |
| **Domain 1: Research team and reﬂexivity** | | | |
| *Personal Characteristics* | | | |
| **1** | Interviewer/facilitator | Which author/s conducted the interview or focus group? | Methods – Patient and Public Involvement and Engagement |
| **2** | Credentials | What were the researcher’s credentials? E.g., PhD, MD | Title page |
| **3** | Occupation | What was their occupation at the time of the study? | Title page  Methods – Patient and Public Involvement and Engagement |
| **4** | Gender | Was the researcher male or female? | See: Albury, C et al., Gender in the consolidated criteria for reporting qualitative research (COREQ) checklist, International Journal for Quality in Health Care, 33, 4, 2021 |
| **5** | Experience and training | What experience or training did the researcher have? | Methods – Patient and Public Involvement and Engagement |
| *Relationship with participants* | | | |
| **6** | Relationship established | Was a relationship established prior to study commencement? | Methods – Patient and Public Involvement and Engagement |
| **7** | Participant knowledge of the interviewer | What did the participants know about the researcher? e.g. personal goals, reasons for doing the research | Methods – Sampling Procedure and Recruitment |
| **8** | Interviewer characteristics | What characteristics were reported about the inter viewer/facilitator? e.g. Bias, assumptions, reasons and interests in the research topic | Methods – Patient and Public Involvement and Engagement  Methods – Data Analysis |
| **Domain 2: study design** | | | |
| *Theoretical framework* | | | |
| **9.** | Methodological orientation and Theory | What methodological orientation was stated to underpin the study? e.g. grounded theory, discourse analysis, ethnography, phenomenology, content analysis | Methods – Data Analysis |
| *Participant selection* | | | |
| **10.** | Sampling | How were participants selected? e.g. purposive, convenience, consecutive, snowball | Methods - Sampling Procedure and Recruitment |
| **11.** | Method of approach | How were participants approached? e.g. face-to-face, telephone, mail, email | Methods - Sampling Procedure and Recruitment |
| **12.** | Sample size | How many participants were in the study? | Results |
| **13.** | Non-participation | How many people refused to participate or dropped out? Reasons? | Results |
| *Setting* | | | |
| **14.** | Setting of data collection | Where was the data collected? e.g. home, clinic, workplace | Methods – Data Collection |
| **15.** | Presence of non-participants | Was anyone else present besides the participants and researchers? | Methods – Data Collection |
| **16.** | Description of sample | What are the important characteristics of the sample? e.g. demographic data, date | Results |
| *Data collection* | | | |
| **17.** | Interview guide | Were questions, prompts, guides provided by the authors? Was it pilot tested? | Methods - Patient and Public Involvement and Engagement |
| **18.** | Repeat interviews | Were repeat interviews carried out? If yes, how many? | Methods – Data Collection |
| **19.** | Audio/visual recording | Did the research use audio or visual recording to collect the data? | Methods – Data Collection |
| **20.** | Field notes | Were ﬁeld notes made during and/or after the interview or focus group? | Methods – Data Collection |
| **21.** | Duration | What was the duration of the interviews or focus group? | Methods – Data Collection |
| **22.** | Data saturation | Was data saturation discussed? | Discussion |
| **23.** | Transcripts returned | Were transcripts returned to participants for comment and/or correction? | Methods – Data Collection |
| **Domain 3: analysis and ﬁndings** | | | |
| *Data analysis* | | | |
| **24.** | Number of data coders | How many data coders coded the data? | Methods – Data Analysis |
| **25.** | Description of the coding tree | Did authors provide a description of the coding tree? | Methods – Data Analysis |
| **26.** | Derivation of themes | Were themes identiﬁed in advance or derived from the data? | Methods – Data Analysis |
| **27.** | Software | What software, if applicable, was used to manage the data? | Methods – Data Analysis |
| **28.** | Participant checking | Did participants provide feedback on the ﬁndings? | Methods – Data Collection |
| *Reporting* | | | |
| **29.** | Quotations presented | Were participant quotations presented to illustrate the themes/ﬁndings? Was each quotation identiﬁed? e.g. participant number | Results |
| **30.** | Data and ﬁndings consistent | Was there consistency between the data presented and the ﬁndings? | Results |
| **31.** | Clarity of major themes | Were major themes clearly presented in the ﬁndings? | Results |
| **32.** | Clarity of minor themes | Is there a description of diverse cases or discussion of minor themes? | Results |

† Tong A, Sainsbury P, Craig J, et al. Consolidated criteria for reporting qualitative research (COREQ): a 32-item checklist for interviews and focus groups. *International Journal for Quality in Health Care*. 2007; 19(6): 349 – 357.

**S2. Topic Guides**

**For people with SMI**

1. In your own words, please describe your experience and journey regarding weight gain?
2. What do you think of support groups like WeightWatchers or Slimming World?

- 1. Have you joined any of these groups? What aspects did you find appealing or unappealing about them?

1. What are your thoughts on the role of social prescribing link workers in healthcare? These are people who work in the NHS who help patients access support groups like WeightWatchers.
   1. Have you heard of social prescribing link workers?
   2. Have you worked with social prescribing link workers? If yes, what aspects did you appreciate or find unfavourable about your interaction with them?
2. In what ways, if any, do you think that social prescribing link workers could help you to lose weight?
   1. Do you think it should be someone else’s responsibility? If yes, who?
3. It might be possible to connect you with a social prescribing link worker to assist you in your weight loss journey? What are your thoughts on this approach?
4. In what ways, if any, do you think that a weekly check-in would support your weight loss journey?
5. Do you have any doubts or queries on what we just said?
6. Finally, is there anything else that you’d like to add or that you think we might have missed?

**For link- and health promotion workers**

1. In your own words, please describe your role as a social prescribing link worker?
   1. Could you describe your activities and responsibilities on a week-to-week basis?
2. What are your thoughts on helping patients in accessing slimming club groups like WeightWatchers or Slimming World?
3. What is your experience of working with people who have been diagnosed with SMI (incl. schizophrenia or bipolar) in healthcare?
   1. What aspects did you find rewarding or challenging in your encounters together?
   2. Do you have any training or experience in working with people with SMI?
   3. If not, what knowledge gaps would you like to be filled?
4. In what ways, if any, do you think that you could help people with SMI to lose weight?
   1. Do you think it should be someone else’s responsibility. If yes, who?
5. How do you feel about working with people who have SMI?
   1. For example, help them to enrol with their slimming club group, join them in their first session, and check-in with them once a week?
6. In what ways, if any, do you think that a weekly check-in would support their weight loss journey?
   1. What questions should be asked and what would the structure of the check-in be?
7. Do you have any doubts or queries on what we just said?
8. Finally, is there anything else that you’d like to add or that you think we might have missed?

**S3. Additional Quotes**

| **Theme & Sub-theme** | **Quotation** | **Participant** |
| --- | --- | --- |
| **1. The view of link workers** | | |
| **1.1 Overcome initial fear and anxiety** | ... you know a child that’s doing something for the first time on their own. And, so why, why, when somebody’s world’s been turned upside down and they’ve been thrust into chaos would you, would you not think that they would need support to do normal everyday things. | Janice, 54-years old female, diagnosed with schizophrenia |
| **1.2 Foster a sense of belonging** | When somebody normally believed in me in my life, I was able to go to university and I was able to do certain things, so, so from my lived experience I think belief is a really big thing in instilling that, especially from somebody who’s a link worker. | Janice, 54-years old female, diagnosed with schizophrenia |
|  | And then when I got in there I was really anxious like cos I thought, ‘I'm out of place here | Janice, 54-years old female, diagnosed with schizophrenia |
| **1.3 Offer practical support** | she was going to come and knock on my door and say, “Right I’m picking you up at 10 o’clock on Monday morning and we’re going swimming, and I need to know exactly why you’re not doing that.” If she did that then it would’ve been alright, she’d have been good, but she was just signposting. | Louise, 57-years old, female, diagnosed with bipolar |
| **2. Expectations of link workers** | | |
| **2.1 Be an accountable space** | […] if you put on weight or you lost weight then somebody’s going like, “Well done, oh don’t worry you’ve not lost any this week,” that confidence in that somebody kind of, somebody being there with you to kind of shift you along a bit, so you’re not, I suppose it’s that bit you’re not on your own. | Louise, 57-years old, female, diagnosed with bipolar |
|  | I need my Dad to come and say, “Right, you’re going swimming and you’re going now, and you’re doing it at that time.” And a social prescriber or a link worker if, if they did that it would probably have a really good outcome. | Louise, 57-years old, female, diagnosed with bipolar |
|  | … because for me success can only be achieved [um] in that way if, if, if you’ve got trust in that individual and they’ve got your best interests at heart | Janice, 54-years old female, diagnosed with schizophrenia |
|  | So to me it’s not a short-term relationship, it’s a long-term. Maybe go to a monthly after a year, I, I would like, I need the motivation of a, of a peer, of someone who I’ve got a rapport with, who’s someone I trust and I know who’s got, their intentions are, are honourable for me | Janice, 54-years old female, diagnosed with schizophrenia |
| **2.2 Be personalised and culturally competent** | People can sometimes feel a bit, “Well then I can’t, I will come back for another two weeks and then I’ll disappear again.” You know that makes it a bit difficult to support someone, regardless of what you’re trying to help them with, if there isn’t that flexibility or kind of [um] commitment to help or desire, in a way desire to understand what their needs might be and how to help them, then it might be very difficult to get them to engage, not by choice, but just they, they just wouldn’t be able to engage | Jennifer, 39-years old, female, link worker |
| **3. Challenges for link workers** | | |
| **3.1 Offer specialised knowledge and training** | It’s somebody that is part of the community and somebody that actually has to come and you know, is doing their job and is supporting us psychologically… I know that [um] they are the right type of people because they understand what’s going on all around us, and quite surprisingly I’d probably say that they’d probably be fantastic as link workers themselves or [um] social prescribers, because they have the social community background. | Adnan, 26-years old, male, with schizoaffective disorder |
|  | I think maybe they need, well they definitely need training within mental health, so of some description, and about medications and the effects of the medications… you know I’m not a gorgy eater, but the problem is, is that my medication is, is making me eat, the side effects of medication. | Adnan, 26-years old, male, with schizoaffective disorder |
| **3.2 Define the role of link workers** | So as long as we’re still empowering them [um] you know I’ll stay in contact, but because we’ve got that flexibility, I think it enables us to build a rapport with some people that might struggle with other services because they’ve got limited time... | Clara, 57 years old, female, link worker |
| **3.3 Address capacity limitations** | So yeah, I’m open to taking people to those groups, [um] if that’s something they need. [Um] But we’ve got limited resources to, to transport people, you know and time I guess, [um] and, you know we can’t be a taxi service, they’ve got to be able to do these things independently. | Clara, 57 years old, female, link worker |

† Participant names are pseudonyms.

End of Supplementary Data
